# Normative values of non-invasively assessed RV function and pulmonary circulation coupling for pre-participation screening derived from 497 male elite athletes

**DOI:** 10.1007/s00392-022-02099-8

**Published:** 2022-09-14

**Authors:** Pascal Bauer, Khodr Tello, Lutz Kraushaar, Oliver Dörr, Stanislav Keranov, Faeq Husain-Syed, Holger Nef, Christian W. Hamm, Astrid Most

**Affiliations:** 1 Department of Cardiology and Angiology, Justus-Liebig-University Giessen, 35390 Giessen, Germany; 2 Department of Internal Medicine, Member of the German Center for Lung Research, Universities of Giessen and Marburg Lung Center, Justus-Liebig-University Giessen, Giessen, Germany; 3 Adiphea GmbH, Werbach, Germany; 4 Department of Cardiology, Kerckhoff Clinic GmbH, Bad Nauheim, Germany

**Keywords:** Right ventricle, Elite athletes, Athlete’s heart, TAPSE/SPAP, Exercise-induced cardiac remodeling

## Abstract

**Background:**

Reference values for right ventricular function and pulmonary circulation coupling were recently established for the general population. However, normative values for elite athletes are missing, even though exercise-related right ventricular enlargement is frequent in competitive athletes.

**Methods:**

We examined 497 healthy male elite athletes (age 26.1 ± 5.2 years) of mixed sports with a standardized transthoracic echocardiographic examination. Tricuspid annular plane excursion (TAPSE) and systolic pulmonary artery pressure (SPAP) were measured. Pulmonary circulation coupling was calculated as TAPSE/SPAP ratio. Two age groups were defined (18–29 years and 30–39 years) and associations of clinical parameters with the TAPSE/SPAP ratio were determined and compared for each group.

**Results:**

Athletes aged 18–29 (*n* = 349, 23.8 ± 3.5 years) displayed a significantly lower TAPSE/SPAP ratio (1.23 ± 0.3 vs. 1.31 ± 0.33 mm/mmHg, *p* = 0.039), TAPSE/SPAP to body surface area (BSA) ratio (0.56 ± 0.14 vs. 0.6 ± 0.16 mm*m^2^/mmHg, *p* = 0.017), diastolic blood pressure (75.6 ± 7.9 vs. 78.8 ± 10.7 mmHg, *p* < 0.001), septal wall thickness (10.2 ± 1.1 vs. 10.7 ± 1.1 mm, *p* = 0.013) and left atrial volume index (27.5 ± 4.5 vs. 30.8 ± 4.1 ml/m^2^, *p* < 0.001), but a higher SPAP (24.2 ± 4.5 vs. 23.2 ± 4.4 mmHg, *p* = 0.035) compared to athletes aged 30–39 (*n* = 148, 33.1 ± 3.4 years). TAPSE was not different between the age groups. The TAPSE/SPAP ratio was positively correlated with left ventricular stroke volume (*r* = 0.133, *p* = 0.018) and training amount per week (*r* = 0.154, *p* = 0.001) and negatively correlated with *E*/*E*′ lat. (*r* = −0.152, *p* = 0.005).

**Conclusion:**

The reference values for pulmonary circulation coupling determined in this study could be used to interpret and distinguish physiological from pathological cardiac remodeling in male elite athletes.

## Introduction

Functional and structural changes of the right and left ventricle, known as the “athlete´s heart”, have been described in elite athletes performing mixed and endurance sports disciplines [1–3]. These physiological adaptations are considered beneficial to generate high performance levels [4]. However, several studies indicated a higher hemodynamic load for the right ventricle (RV) and the pulmonary circulation unit during exercise compared to the left ventricle and LV-arterial coupling [5, 6]. It was even hypothesized that these may lead to a disproportioned remodeling of the RV, resulting in an increased risk for RV cardiomyopathy and arrhythmias in highly trained athletes [7–10].

In this context, the pivotal role that the RV and the pulmonary circulation coupling play in many different cardiopulmonary conditions has been emphasized [11–13]. Furthermore, pulmonary circulation coupling was identified as a load-independent indicator of RV function allowing the detection of RV dysfunction before the RV ejection fraction decreases [14].

Recently, new non-invasive echocardiographic methods have emerged as surrogates for pulmonary circulation coupling and were successfully validated against invasive measurements [15, 16]. The ratio of tricuspid annular plane systolic excursion (TAPSE) to pulmonary artery systolic pressure (SPAP) was hence proposed as a simple echocardiographic indicator of pulmonary circulation coupling for routine clinical application [16, 17].

In contrast to the age changes in LV-arterial coupling that are characterized by a proportionally stiffening of both LV and the arterial vascular tree [18, 19], a relatively preserved relationship between TAPSE and SPAP was found across different age groups among healthy subjects and respective reference values have been published [20].

However, these parameters have not been evaluated in elite athletes, until now, and reference values for athletes are missing. We, therefore, conducted this echocardiographic study in two different age groups of male elite athletes performing different mixed sports to deliver first reference values and to gain more insights into the functional and structural adaptations of the pulmonary circulation coupling in highly trained individuals. Since professional athletes typically display functional and morphological parameters at the high end of the healthy spectrum, we did not expect to discover significant differences when compared to healthy adults of comparable age. Accordingly, the present study aimed to describe the relationship between structural and functional adaptations to exercise and compare them to the published reference values for the general population [20]. Furthermore, we investigated the influence of clinical (age, body surface area (BSA)), sports related (training history and training amount per week) and echocardiographic factors (*E*/*A*, LV stroke volume, *E*/*E*′, heart rate) on pulmonary circulation coupling in these athletes. We hence hypothesized that our examined male elite athletes would not show significant differences from the proposed reference values for healthy male individuals nor between the two examined age groups.

## Methods

### Study design

This was a single-center, cross-sectional study conducted at the university hospital Giessen involving professional athletes during the routine pre-season medical monitoring program of the first German handball and basketball divisions and the second German handball and ice-hockey divisions. Data were collected in July and August of the years 2017–2021 after a 6-week competition-free interval. Only male athletes aged 18–39 were included in the study. Due to the overwhelming male athlete population service by our center, we did not have enough elite female athletes to investigate potential gender differences.

All participants provided their written informed consent. Subjective health status, medication, nutrition supplementation, amount of training, and history of training were assessed by questionnaire. Only individuals free of underlying cardiovascular diseases and medication were included. All tests were conducted at least 3 h post-prandially, and subjects refrained from exercise for at least 36 h prior to the test.

The local ethics committee of the university of Giessen approved the study protocol (AZ 15/17). The study was performed in accordance with the ethical standards laid down in the Declaration of Helsinki and its later amendments.

### Study population

The participants were 497 healthy, injury-free Caucasian male professional athletes of different nationalities performing different mixed sports (Handball, Basketball, Ice-hockey). All participants included were non-smokers and none took medication or supplements on a regular basis. All individuals were subjected to a physical examination, 12-lead electrocardiogram (ECG), blood pressure measurements at rest and transthoracic echocardiography. Age, height, weight, and body mass index were determined. Body surface area was calculated using the formula of DuBois. Athletes then were divided into two age groups (18–29 years and 30–39 years) and statistical analyses were performed. The division in two age groups was made to compare the results with the reference population [20] and to gain insights into age changes of the evaluated parameters.

### Blood pressure measurement at rest

Resting brachial blood pressure was measured using a validated automatic device based on a standard sphygmomanometer technique (Boso clinicus, Bosch + Sohn GmbH & Co. KG, Germany). The cuff used for measurements was adjusted to the individual´s arm circumference. Measurements were performed by a trained research associate on both arms in a sitting position after a resting period of 5 min and repeated after 2 min. The average blood pressure (BP) was used for statistical analyses. Athletes with a mean BP ≥ 140 mmHg systolic or ≥ 90 mmHg diastolic were excluded from the study.

### Echocardiography

All athletes were examined by standard transthoracic echocardiography administered by an experienced cardiologist according to the current recommendations [21, 22] using a Philips cx50 echocardiography system (Philips, Eindhoven, the Netherlands) with the participant placed in a left lateral supine position. Standard measurements of cardiac dimensions, contractility and diastolic function were obtained. Each parameter was assessed in three to five consecutive cardiac cycles, and mean values were used for data recording and analysis.

Left ventricular (LV) wall thicknesses and diameters were evaluated in the parasternal long-axis view at the level of mitral valve coaptation. Furthermore, volumes and ejection fraction were determined using Simpson´s biplane-method. LV stroke volume was calculated as the product of LV outflow tract area and outflow tract time-velocity integral. RV stroke volume was accordingly calculated as the product of RV outflow tract area and outflow tract time-velocity integral.

LV mass was calculated using the Devereux formula and indexed to body surface area to obtain the LV mass index (LVMI). LV hypertrophy was defined as a LVMI > 115 g/m^2^. Left atrial volume index (LAVI) was obtained by the area-length method.

Peak tricuspid regurgitant velocity (TRV) was measured from the spectral profile of the tricuspid regurgitation jet in the right ventricular inflow projection of the parasternal short-axis view or the apical four-chamber view. Pulmonary artery systolic pressure (SPAP) was then calculated based on the simplified Bernoulli equation applied to TRV by adding a value of right atrial pressure as measured by inferior Vena Cava respiratory index to the systolic trans-tricuspid gradient. SPAP was assumed to equate the right ventricular systolic pressure in the absence of pulmonic stenosis and/or right ventricular outflow tract obstruction.

Tricuspid annular plane systolic excursion (TAPSE) was measured from the four-chamber views by placing an M-mode cursor through the tricuspid annulus, measuring the excursion distance between end-diastole and end-systole in millimeter (mm).

The TAPSE/SPAP ratio was calculated as TAPSE divided by SPAP for each participant. To avoid confounding effects of anthropometric differences, and to ensure comparison to the published reference values [20], we also indexed the TAPSE/SPAP ratio by body surface area.

### Statistical analysis

Descriptive analyses were carried out on all study variables for the total sample and separated by age status (18–29 years and ≥ 30–39 years). All data are presented as mean ± standard deviation (SD). The Shapiro–Wilk test was used to determine normal distribution. If the data were determined to have a skewed distribution, all analyses were performed on normalized data. Between-group comparisons were made using independent sample *t* tests. Bivariate relations were analyzed using the Spearman correlation coefficient. The Pearson partial correlation test was used to assess the relationship between clinically relevant variables such as age, body mass index (BMI), body surface area (BSA), training history, amount of training per week, LV stroke volume, heart rate, *E*/*A* and *E*/*E*′.

Then, multiple regression analyses were performed to test the weight of these variables within and across two age classes. For this, we divided the cohort into two groups according to their age class (18–29 years and 30–39 years).

A quantile regression analyses was used to describe quantiles (0.05, 0.95, and all deciles) of SPAP, TAPSE, TAPSE/SPAP and TAPSE/SPAP/BSA as reference values for these parameters in professional male athletes.

Two-tailed significance level was set at *p* < 0.05 for all measurements. All statistical analyses were performed using the IBM SPSS Statistics for Macintosh, Version 27.0 (IBM Corp., Armonk, NY, USA).

## Results

### Cohort characteristics

All 497 male elite athletes included in the study were participants in mixed team sports disciplines that are characterized by a high-intensity level (handball, ice-hockey, and basketball) [4]. The mean age of the participants was age 26.1 ± 5.2 years with a mean height of 189.5 ± 7.6 cm and a mean weight of 92.8 ± 11 kg, resulting in mean body mass index of 25.9 ± 1.9 kg/m^2^. The probands were experienced athletes who had participated in professional training for 9.4 ± 4.1 years with a current mean training time of 18.7 ± 3.2 h per week.

There were no significant differences regarding height, weight, body mass index and body surface area or amount of training per week between athletes aged 18–29 and those aged 30–39 years. As expected, the older group had a longer professional training history than the younger group (7.15 ± 3.6 vs. 15.8 ± 3.1 years, *p* < 0.001). The clinical characteristics, anthropometric data, and specific training data are displayed in detail in Table 1.

**Table 1:** Clinical characteristics of the included athletes, divided by age groups

	Male elite athletes		*p* value
	Age 18–29	Age 30–39	
Number	349	148	
Age (years)	23.8 ± 3.5	33.1 ± 3.4	** < 0.001**
Height (cm)	189.7 ± 7.7	188.9 ± 7.2	0.321
Body weight (kg)	93 ± 11.3	92.5 ± 9.9	0.660
Body mass index (kg/m^2^)	25.8 ± 2.1	25.8 ± 1.3	0.588
Body surface area (m^2^)	2.21 ± 0.17	2.20 ± 0.16	0.552
Training history (years)	7.15 ± 3.6	15.8 ± 3.1	** < 0.001**
Training per week (h)	19 ± 3.2	19 ± 2.9	0.760
Systolic blood pressure (mmHg)	123.8 ± 10.3	123.8 ± 10.7	0.985
Diastolic blood pressure (mmHg)	75.6 ± 7.9	78.8 ± 6.8	** < 0.001**
Mean arterial blood pressure (mmHg)	91.7 ± 7	93.8 ± 6.4	**0.002**
Resting heart rate (/min)	58.1 ± 10.9	57.7 ± 9.8	0.739

Bold values denote statistical significance at the *p* < 0.05 level

### Blood pressure measurements

The mean systolic and diastolic BP values of the entire study cohort was 123.8 ± 16.4 mmHg and 76.4 ± 7.7 mmHg, respectively. There were no significant differences in systolic BP between athletes aged 18–29 (123.8. ± 10.7 mmHg) and those aged 30–39 (123.8 ± 10.3 mmHg). In contrast, younger athletes (18–29 years) displayed a significantly (*p* < 0.01) lower diastolic BP (76.6 ± 7.9 mmHg) and mean BP (91.7 ± 7 mmHg) compared to their older (30–39 years) peers (78.8 ± 6.8 mmHg diastolic BP and 93.8 ± 6.4 mmHg mean BP, respectively). None of the included athletes had a systolic BP ≥ 140 mmHg or diastolic BP ≥ 90 mmHg.

### Echocardiographic characteristics

Echocardiographic characteristics of the two age groups are summarized in Table 2. The two age groups differed significantly in the left atrial diameter (*p* = 0.013), left atrial volume index (*p* < 0.001) and septal wall thickness (*p* < 0.001), with older athletes (30–39 years) displaying higher values for each parameter.

**Table 2:** Echocardiographic findings of the included athletes included, divided by age groups

	Male elite athletes		*p* value
	Age 18–29	Age 30–39	
*Echocardiographic parameters*			
LV ejection fraction (%)	66.6 ± 4.6	67.1 ± 4.7	0.320
LV stroke volume (ml)	91.4 ± 18.9	92.6 ± 16	0.567
LV enddiastolic diameter (mm)	53.6 ± 3.9	54.2 ± 3.3	0.123
LV endsystolic diameter (mm)	33.4 ± 3.5	33.7 ± 3.5	0.428
Left atrial diameter (mm)	37.2 ± 3.3	38.1 ± 2.9	**0.013**
Left atrial volume index (ml/m^2^)	27.5 ± 4.5	29.3 ± 4.1	** < 0.001**
Septal wall thickness (mm)	10.2 ± 1.1	10.7 ± 1.1	** < 0.001**
Inferior wall thickness (mm)	9.8 ± 1	10 ± 0.97	0.134
LV mass index (g/m^2^)	88.7 ± 24	90.2 ± 33.8	0.490
*E*/*A* ratio	1.85 ± 0.45	1.75 ± 0.41	0.067
*E*/*E*′ lateral	5.45 ± 1.35	5.34 ± 1.34	0.532
*E*/*E*′ medial	6.79 ± 1.28	6.98 ± 1.26	0.169
*E*/*E*′ average	6.13 ± 1.08	6.33 ± 1.11	0.231
RV diameter 1 (mm)	38.8 ± 5.2	40.2 ± 4.8	0.094
RVOT PLAX (mm)	30.8 ± 2.2	31.2 ± 1.7	0.138
RV stroke volume (mm)	88.5 ± 15.2	90.2 ± 17.5	0.102
Fractional area change (%)	54.1 ± 8.3	52.8 ± 6.4	0.074
RV s’ (cm/s)	14.4 ± 3.8	13.8 ± 4.4	0.086
TAPSE (mm)	28.9 ± 4.2	29.4 ± 4.8	0.308
SPAP (mmHg)	24.2 ± 4.5	23.2 ± 4.4	**0.035**
TASPSE/SPAP (mm/mmHg)	1.23 ± 0.3	1.31 ± 0.33	**0.039**
TAPSE/BSA (mm/m^2^)	13.1 ± 1.9	13.5 ± 2.4	0.117
TAPSE/SPAP/BSA (mm*m^2^/mmHg)	0.56 ± 0.14	0.60 ± 0.16	**0.017**

Bold values denote statistical significance at the *p* < 0.05 level

*BMI*  body mass index; *LV*  left ventricle; *PLAX* parasternal long axis view; *RV* right ventricle; *RVOT* right ventricular outflow tract; *SPAP* systolic pulmonary artery pressure; *TAPSE* tricuspid annular plane systolic excursion

In addition, SPAP was lower (*p* = 0.035) in older athletes compared to the younger group. As TAPSE was not different (*p* = 0.308) between the two groups, we observed a significant difference in the TAPSE/SPAP ratio (*p* = 0.039) and the TAPSE/SPAP/BSA (*p* = 0.017), with older athletes (30–39 years) having higher values.

### Clinical and echocardiographic predictors of TAPSE, SPAP, TAPSE/SPAP and TAPSE/SPAP/BSA

TAPSE was positively correlated with age (*r* = 0.104, *p* = 0.028), BMI (*r* = 0.155, *p* = 0.001) and the LV stroke volume (*r* = 0.224, *p* < 0.001) in the whole study cohort. There were positive correlations between SPAP and BMI (*r* = 0.140, *p* = 0.004), BSA (*r* = 0.124, *p* = 0.010) and *E*/*E*′ lat. (*r* = 0.197, *p* < 0.001), whereas a negative correlation with training per week (*r* = −0.114, *p* = 0.018) was apparent.

The TAPSE/SPAP ratio as surrogate parameter of right ventricular-pulmonary coupling was positively correlated with training duration per week (*r* = 0.154, *p* = 0.001), LV stroke volume (*r* = 0.133, *p* = 0.018) and negatively correlated with *E*/*E*′ lat. (*r* = −0.152, *p* = 0.005). This negative correlation remained after adjustment for BSA (*r* = −0.156, *p* = 0.004) as displayed by the TAPSE/SPAP/BSA.

Pearson´s partial correlation test results for the clinical and echocardiographic parameters are presented in Table 3.

**Table 3:** Pearson correlation coefficients for the association between clinical characteristics and pulmonary circulating coupling parameters obtained by echocardiography

	TAPSE		SPAP		TAPSE/SPAP		TAPSE/SPAP/BSA	
	*r*	*p*	*r*	*p*	*r*	*p*	*r*	*p*
Age (y)	0.104	**0.028**	−0.008	0.874	0.060	0.214	0.061	0.210
BMI (kg/m^2^)	0.155	**0.001**	0.140	**0.004**	−0.023	0.636	−0.192	** < 0.001**
BSA (m^2^)	0.043	0.381	0.124	**0.010**	0.030	0.541	−0.275	** < 0.001**
Training history (y)	0.075	0.113	0.077	0.113	−0.022	0.653	−0.034	0.481
Training per week (h)	0.115	0.016	−0.114	**0.018**	0.154	**0.001**	0.063	0.192
LV stroke volume (ml)	0.224	** < 0.001**	0.024	0.664	0.133	**0.018**	0.023	0.682
Heart rate (/min)	−0.012	0.796	0.070	0.146	−0.058	0.231	−0.068	0.160
*E*/*A*	−0.023	0.679	0.001	0.982	−0.018	0.755	0.007	0.903
*E*/*E*′ lat	−0.009	0.864	0.197	** < 0.001**	−0.152	**0.005**	−0.156	**0.004**
*E*/*E*′med	0.006	0.893	0.001	0.992	0.033	0.492	0.057	0.243
*E*/*E*′average	0.007	0.872	0.098	0.462	−0.072	0.102	−0.086	0.098

Bold values denote statistical significance at the *p* < 0.05 level

*BMI* body mass index; *BSA* body surface area; *LV* left ventricle; *SPAP* systolic pulmonary artery pressure; *TAPSE* tricuspid annular plane systolic excursion

### Associations of TAPSE, SPAP, TAPSE/SPAP and TAPSE/SPAP/BSA with clinical and echocardiographic predictors by age groups

Multiple regression analyses were performed to identify independent determinants for TAPSE, SPAP, TAPSE/SPAP and TAPSE/SPAP/BSA in the two age groups. We, therefore, determined a statistical model for each dependent parameter. In the statistic model, we used age, BMI, BSA, training history, training duration per week, LV stroke volume, heart rate, E/A and *E*/*E*′ average as independent predictors of the respective dependent variable. The respective results are presented in Table 4.

**Table 4:** Multiple regression analyses with pulmonary circulating coupling parameters as dependent variables, displayed for the two age groups

Age 18–29	TAPSE		SPAP		TAPSE/SPAP		TAPSE/SPAP/BSA	
	*R*^2^ 0.158, *p* = 0.001		*R*^2^ 0.193, *p* < 0.001		*R*^2^ 0.081, *p* = 0.144		*R*^2^ 0.193, *p* < 0.001	
	*b*	*p*	*b*	*p*	*b*	*p*	*b*	*p*
Age (y)	0.007	0.963	0.024	0.875	−0.101	0.527	−0.068	0.649
BMI (kg/m^2^)	−0.115	0.217	0.019	0.833	−0.118	0.214	−0.126	0.157
BSA (m^2^)	0.387	** < 0.001**	0.186	**0.039**	0.087	0.361	−0.262	**0.004**
Training history (y)	0.088	0.576	0.263	0.090	−0.091	0.582	−0.105	0.498
Training per week (h)	−0.103	0.227	−0.079	0.337	0.004	0.961	0.007	0.931
LV SV (ml)	0.134	0.089	0.113	0.142	0.008	0.918	0.008	0.913
Heart rate (/min)	0.016	0.831	0.122	0.100	−0.087	0.361	−0.073	0.325
E/A	0.012	0.867	0.093	0.198	−0.085	0.269	−0.090	0.212
*E*/*E*′ lat	−0.102	0.215	0.073	0.369	−0.145	0.094	−0.133	0.102
*E*/*E*′ med	0.014	0.862	0.022	0.781	0.018	0.835	0.029	0.718
*E*/*E*′ average	0.034	0.640	0.052	0.562	−0.086	0.367	−0.052	0.517

Age 30–39	TAPSE		SPAP		TAPSE/SPAP		TAPSE/SPAP/BSA	
	*R*^2^ 0.291, *p* = 0.034		*R*^2^ 0.215, *p* = 0.184		*R*^2^ 0.175, *p* = 0.365		*R*^2^ 0.226, *p* = 0.148	
	*b*	*p*	*b*	*p*	*b*	*p*	*b*	*p*
Age (y)	0.064	0.717	0.066	0.722	0.062	0.743	0.068	0.712
BMI (kg/m^2^)	0.120	0.463	0.236	0.174	−0.039	0.825	−0.024	0.889
BSA (m^2^)	−0.025	0.895	−0.110	0.586	−0.016	0.940	−0.293	0.146
Training history (y)	−0.310	0.113	−0.268	0.191	−0.060	0.772	−0.075	0.712
Training per week (h)	−0.054	0.670	0.259	0.055	−0.217	0.114	−0.197	0.137
LV SV (ml)	0.255	0.066	−0.265	0.069	0.361	**0.017**	0.349	**0.017**
Heart rate (/min)	0.272	0.057	0.001	0.998	0.174	0.253	0.155	0.292
E/A	0.098	0.479	−0.151	0.301	0.210	0.163	0.202	0.163
*E*/*E*′ lat	0.351	**0.024**	0.127	0.427	0.108	0.509	0.116	0.466
*E*/*E*′med	−0.149	0.347	−0.053	0.749	−0.039	0.818	−0.051	0.759
*E*/*E*′average	0.212	0.178	0.023	0.614	0.062	0.782	0.023	0.686

Bold values denote statistical significance at the *p* < 0.05 level

*BMI*  body mass index; *LV*  left ventricle; *SPAP*  systolic pulmonary artery pressure; *SV*  stroke volume; *TAPSE*  tricuspid annular plane systolic excursion

The model was not able to predict TAPSE, SPAP, TAPSE/SPAP and TAPSE/SPAP/BSA in both groups. In younger athletes (18–29 years), BSA was positively associated with TAPSE (β = 0.387, *p* < 0.001), SPAP (β = 0.186, *p* = 0.039) and negatively associated with TAPSE/SPAP/BSA (β = −0.262, *p* = 0.004). In the group of older athletes (30–39 years), LV stroke volume was positively associated with TAPSE/SPAP (β = 0.361, *p* = 0.017) and TAPSE/SPAP/BSA (β = 0.349, *p* = 0.017).

### Reference values for TAPSE, SPAP, TAPSE/SPAP, TAPSE/SPAP/BSA according to age group

Age-specific normative values for TAPSE, SPAP, TAPSE/SPAP and TAPSE/SPAP/BSA were calculated and displayed in Table 5 with respective percentiles. We calculated respective percentiles including 5th and 95th percentiles for each parameter and age group. The determination of age groups was chosen to ensure comparison to the published reference values [20]. For male elite athletes aged 18–29 years, the 5th percentile for TAPSE/SPAP would be 0.82 mm/mmHg and the 95th percentile 1.84 mm/mmHg. The corresponding values for athletes aged 30–39 years would be 0.84 and 1.94 mm/mmHg, respectively.

**Table 5:** Normative values of the pulmonary circulating coupling parameters, given in percentiles and displayed for the two age groups

Percentile	Age 18–29				Age 30–39			
	TAPSE (mm)	SPAP (mmHg)	TAPSE/PASP (mm/mmHg)	TAPSE/SPAP/BSA (mm*m^2^/mmHg)	TAPSE (mm)	SPAP (mmHg)	TAPSE/PASP (mm/mmHg)	TAPSE/SPAP/BSA (mm*m^2^/mmHg)
5 th	23	16	0.82	0.37	21.8	16	0.84	0.41
10 th	24	18	0.9	0.41	23	18	0.9	0.43
20 th	25	20	1.0	0.45	26	19	1.0	0.47
30 th	26	22	1.08	0.49	26.5	21	1.11	0.51
40 th	28	23	1.1	0.52	28	22	1.19	0.53
50 th	29	24	1.21	0.55	30	22.5	1.28	0.57
60 th	30	25	1.27	0.59	31	24	1.37	0.62
70 th	31	26	1.37	0.63	31.5	25	1.44	0.67
80 th	32.4	28	1.50	0.67	33	27	1.58	0.74
90 th	35	30	1.67	0.77	35.5	29.1	1.73	0.86
95 th	37	30	1.84	0.87	38	33	1.94	0.90

*BSA* body surface area; *SPAP* systolic pulmonary artery pressure; *TAPSE* tricuspid annular plane systolic excursion

## Discussion

The present study, to our knowledge, represents the first detailed evaluation of the TAPSE/SPAP ratio as a non-invasive surrogate of pulmonary circulation coupling in healthy male elite athletes of two different age groups.

Our most important findings are that

1. the TAPSE/SPAP ratio was significantly higher in athletes aged 30–39 years compared to their younger peers aged 18–29 years;
2. we found higher values of both TAPSE and SPAP in male elite athletes compared to the published reference values for aged-matched healthy men;
3. TAPSE/SPAP ratio was positively correlated with the training amount per week and LV stroke volume.

Understanding the structural and functional adaptations in highly trained athletes is essential for differentiating physiological from pathological remodeling in the context of pre-participation evaluation [2, 4] and, therefore, constitutes a relevant clinical issue. In any individual, cardiac adaptations to exercise are influenced by several factors including training mode, intensity, duration and volume [1, 3, 19, 23–26]. Cardiac remodeling of the right ventricle in athletes was intensively investigated [6, 10, 26–32] and different echocardiographic approaches were proposed to identify pathological changes [1, 3, 33]. However, so far, accepted reference values for athletes exist mainly for morphological echocardiographic measurements as highlighted in recent meta-analyses [28, 32]. Despite the elaborated echocardiographic techniques used [1, 33], right ventricular function and pulmonary circulating coupling were rarely investigated together in athletes and the proposed reference values were mainly derived from small and inhomogeneous study samples and without invasive validation [3].

Recently, the TASPE/SPAP ratio was identified as a powerful and easily applicable echocardiographic surrogate of pulmonary circulating coupling [15, 16]. It has been validated against invasive conductance measurements [15]. Furthermore, its relevance was established for several cardiovascular diseases [17, 34], highlighting the robust reflection of pulmonary circulation coupling in various clinical settings. Moreover, age-dependent reference values for healthy individuals are available [20]. Given the remarkably constant SPAP and pulmonary artery wedge pressure during prolonged and increasing exercise intensity in middle-aged athletes, this indicates a highly compliant pulmonary vasculature that accommodates the increasing hemodynamic load and maintains right ventricular function and pulmonary circulating coupling throughout exercise conditions [30]. In consequence, the assumption of a stable TAPSE/SPAP ratio under exercise conditions in healthy athletes is reasonable, indicating this simple metric as a powerful surrogate of pulmonary circulating coupling in athletes.

In our study, the TASPE/SPAP ratio was calculated slightly lower in male elite athletes of both age groups compared to the published age-specific reference values [20]. Interestingly, we also observed higher values for the TAPSE/SPAP ratio in the older age group (30–39 years), which was mainly attributed to a significantly lower SPAP, as in the reference population [20]. Notably, these non-invasively derived TAPSE/SPAP ratio corresponded well with the invasive measurements reported by Buchan et al. [30], suggesting an augmented cardiovascular functional response in highly trained male athletes that ensures an optimal coupling of both the pulmonary and systemic circulation. The positive correlations of TAPSE/SPAP with training amount per week and the LV stroke volume as well as the negative correlation with the *E*/*E*′ lat. ratio in our examined male elite athletes reveal these parameters as relevant indicators of the respective functional adaptations and are in line with the findings of Vriz et al. [20]. Therefore, the training amount per week, but not training history, was identified in our study as an important additional influencing factor, relevant particularly to athletic populations. Unlike the reference population, we found no association between TAPSE/SPAP and age, BMI or BSA, possibly because populations examined differed. Our cohort comprised young adults with narrow age, BMI and BSA ranges, whereas the population analyzed by Vriz et al. [20] aged 18–74 years and displayed a broader BMI and BSA range.

It is important to consider both morphological and functional RV adaptations to differentiate physiological from pathological remodeling and to understand the relatively constant pulmonary circulation coupling measures that ensure an optimal physiological coupling. Enlarged RV compared to the normal population has been demonstrated in different athletic cohorts and is particularly pronounced in endurance athletes [1, 3, 6, 23–25, 28, 31, 32]. In our study, all measured RV parameters were comparable to the published reference values for healthy male elite athletes [23, 28, 32] with similar age. Unfortunately, so far, there exist no data for athletes regarding the TASPE/SPAP ratio. Hence, our study is the first to evaluate this promising marker in a large and homogenous group of male elite athletes and different age.

Other important factors that must be acknowledged when investigating RV morphological and functional changes in athletes are sex and examination time. Female sex was shown to be related to TAPSE, SPAP and TAPSE/SPAP ratio [20], further to RV structure and performance [35]. In addition, RV contractility in women was found to be higher in women compared to men, especially at younger age [36] and in pulmonary arterial hypertension [37].

A wealth of studies confirmed that intense prolonged exercise is associated with both an acute RV enlargement and a reduction in RV function [7], whereas the LV was mainly unaffected [28, 29, 38]. This phenomenon has been named “cardiac fatigue” and encouraged discussions about detrimental effects of prolonged exercise sessions on RV remodeling [9, 10, 29]. In consequence, as shown for healthy adults during an exercise test, TAPSE/SPAP ratio decreased [27]. Hence, assessment of TAPSE/SPAP should be undertaken under similar conditions. In the study of Vriz et al. [20] and our own study, TAPSE/SPAP was determined under resting conditions with a normal brachial blood pressure.

Pre-participation screenings are usually scheduled at the beginning of the competitive season, offering an opportunity to evaluate the morphology and function of the right ventricle and measure the pulmonary circulation coupling at rest, without the confounding influences of prior prolonged exercise sessions. This very sensitive marker, which was already validated against invasive measurements, could be useful for differentiating physiological from pathological RV remodeling. However, this hypothesis should be investigated in future studies.

### Limitations and strengths

Our study has several limitations. The number of participants limited its statistical power to reveal other associations or to determine diagnostic thresholds. The focus on male elite mixed sports athletes might limit extrapolation of the results to other sport disciplines, to an older age group, or to women. Furthermore, we did not control for diet and body composition. In addition, the TAPSE/SPAP ratio, though validated against invasive measurements, still remains a surrogate parameter to assess pulmonary circulation coupling. However, we included male elite athletes of a narrow age span, free of cardiovascular diseases and medication use, and we controlled for confounders like prior prolonged exercise sessions. Furthermore, the homogenous study cohort and the rigid design of measuring cardiovascular function must be mentioned, which strengthens our analysis.

## Conclusion

This study delivered the first reference values for the TAPSE/SPAP ratio in highly trained male athletes performing mixed-exercise sports. Reference values of TAPSE, SPAP and TAPSE/SPAP derived from our male athletic cohort can be used to interpret changes in athletes and may help to distinguish between physiological and pathological adaptations.

## Data Availability

The manuscript data will not be deposited.
